# Single-incision laparoscopic cholecystectomy *versus* conventional multi-port laparoscopic cholecystectomy: A systematic review, meta-analysis, and meta-regression of randomized controlled trials

**DOI:** 10.12688/f1000research.122102.1

**Published:** 2022-07-06

**Authors:** Reno Rudiman, Ricarhdo Valentino Hanafi, Alma Wijaya

**Affiliations:** 1 Division of Digestive Surgery, Department of General Surgery, School of Medicine, Universitas Padjadjaran, Hasan Sadikin General Hospital, Bandung, 40161, Indonesia; 2 Faculty of Medicine, Pelita Harapan University, Tangerang, 15810, Indonesia

**Keywords:** Cholecystectomy, laparoscopic surgery, meta-analysis, minimal invasive surgery, systematic review

## Abstract

**Background:** Conventional multi-port laparoscopic cholecystectomy (CMLC) has become the current ‘gold standard’ technique in gallbladder disease. Single-incision laparoscopic cholecystectomy (SILC) has gained attention due to its benefits in improving patient cosmetic results and pain reduction. We aim to assess the latest evidence on the feasibility, safety and surgical outcomes of SILC and CMLC.

**Methods:** We conducted searches for randomized controlled trials (RCTs) in PubMed, PubMed Central (PMC), and Europe PMC between December 2011 and 2021. The latest search was conducted in January 2022. We analyzed several outcomes, including perioperative complications, estimated blood loss, operation time, conversion to open surgery, hospital stay, pain score, cosmesis, and days of return to work. Cochrane Risk of Bias (RoB) 2.0 tool was used to evaluate quality of studies. Mantel-Haenszel's formula and Inverse Variance method were conducted to synthesize results. This study was accomplished in accordance with the PRISMA guidelines.

**Results:** A total of 37 studies were eligible, with a total of 2,129 and 2,392 patients who underwent SILC and CMLC. Our study demonstrated a superiority of SILC for the visual analog score (VAS) at six hours post-operation [mean difference (MD) -0.58 (95% CI -1.11, -0.05), p=0.03], cosmesis one-month post-operation [standard MD 2.12 (95% CI 1.10, 3.13), p<0.0001], and cosmesis six months post-operation [standard MD 0.53 (95% CI 0.06, 0.99), p<0.0001]. Meanwhile, SILC showed a longer operation time [MD 10.45 (95% CI 6.74, 14.17), p<0.00001]. In terms of VAS at four time points (4, 8, 12, and 24 hours), perioperative complications, estimated blood loss, conversion to open surgery, hospital stay and days to return to work, SILC did not differ from CMLC.

**Conclusions:** SILC is a safe, feasible and favorable procedure in terms of pain reduction and cosmetic results. The option between both procedures is based on surgeon preferences.

**Registration:** PROSPERO ( CRD42022306532; 23 February 2022).

## Introduction

For decades, conventional multi-port laparoscopic cholecystectomy (CMLC) has been a favored procedure in gall bladder diseases. ^1^ This technique differs from any laparoscopic surgery, which requires advanced technology and skill. The gains in CMLC aggregate the learning curve and surgical aftermaths. ^2^ Many adjustments and modifications are made in CMLC; fundamentally, CMLC consists of four ports. The adaptations may be a reduced port size, such as 10 mm to 5 mm or 5 mm to 2/3 mm, or reduced port numbers. These changes intend to amend patient outcomes in any aspect. ^3^

The curiosity about single-incision laparoscopic cholecystectomy (SILC) has increased in the past years. A single incision implies when many ports are inserted at a single site (umbilicus). Many studies have demonstrated technical difficulty and increasing complications. ^4^^,^^5^ A recent study indicated a feasible and valuable technique with superior cosmetic outcomes, pain reduction, and nil complications. ^6^ We speculate that knowledge about SILC will improve in the following years.

Pros and cons remain disputable in many systematic reviews. ^7^^–^^12^ We would like to update the recent randomized controlled trials (RCTs) regarding these techniques because there has not been a newer systematic review to analyze the latest amendment. The latest meta-analysis by Lyu *et al*., ^12^ determined that a limitation of their study was that only three and four ports of instruments, plus non-RCT were included; thus, our study addresses the limitations of the latest research and expects different outcomes. Our hypothesis is that SILC is superior to CMLC in all aspects. Consequently, this study aimed to assess and evaluate the latest evidence on the feasibility, safety, and surgical outcomes of SILC and CMLC.

## Methods

### Eligibility criteria

We conducted a systematic review and meta-analysis study from clinical trial studies. We registered this systematic review in PROSPERO ( CRD42022306532) on 23 February 2022. Articles were included in this systematic review and meta-analysis if they fulfilled the Population, Intervention, Comparison, Outcomes and Study (PICOS) framework as follows:

• P - Population: Adults aged >18 years old with body mass index (BMI) <35 kg/m ^2^ with uncomplicated gall bladder disease who were eligible to undergo either SILC or CMLC with the American Society of Anesthesiology (ASA) Score I-III. ^13^
• I - Intervention: Patients who underwent SILC to treat their gall bladder diseases.
• C - Comparator: Patients who underwent CMLC as surgical treatment for gall bladder diseases.
• O - Outcomes: Bile duct injury (BDI), bile leakage, gallbladder perforation, wound infection, incisional hernia, total intraoperative complications, total post-operative complications, conversion to open cholecystectomy, operating time, estimated blood loss volume, length of hospital stay, cosmesis (satisfactory quantitative scores) at one month and six months post-operation, days to return to work, and post-operative pain score assessed by a visual analog score (VAS) at five-time points (4, 6, 8, 12, and 24 hours post-procedure).
• S - Study design: Randomized clinical trials

All studies besides original articles (correspondence, letter to editor, or review articles), observational studies (cohort or case-control designs), case series, case report studies, studies reported in a language other than English, research focusing on pregnant women and populations below the age of 18 years were excluded.

### Search strategy and study selection

**Figure 1.**
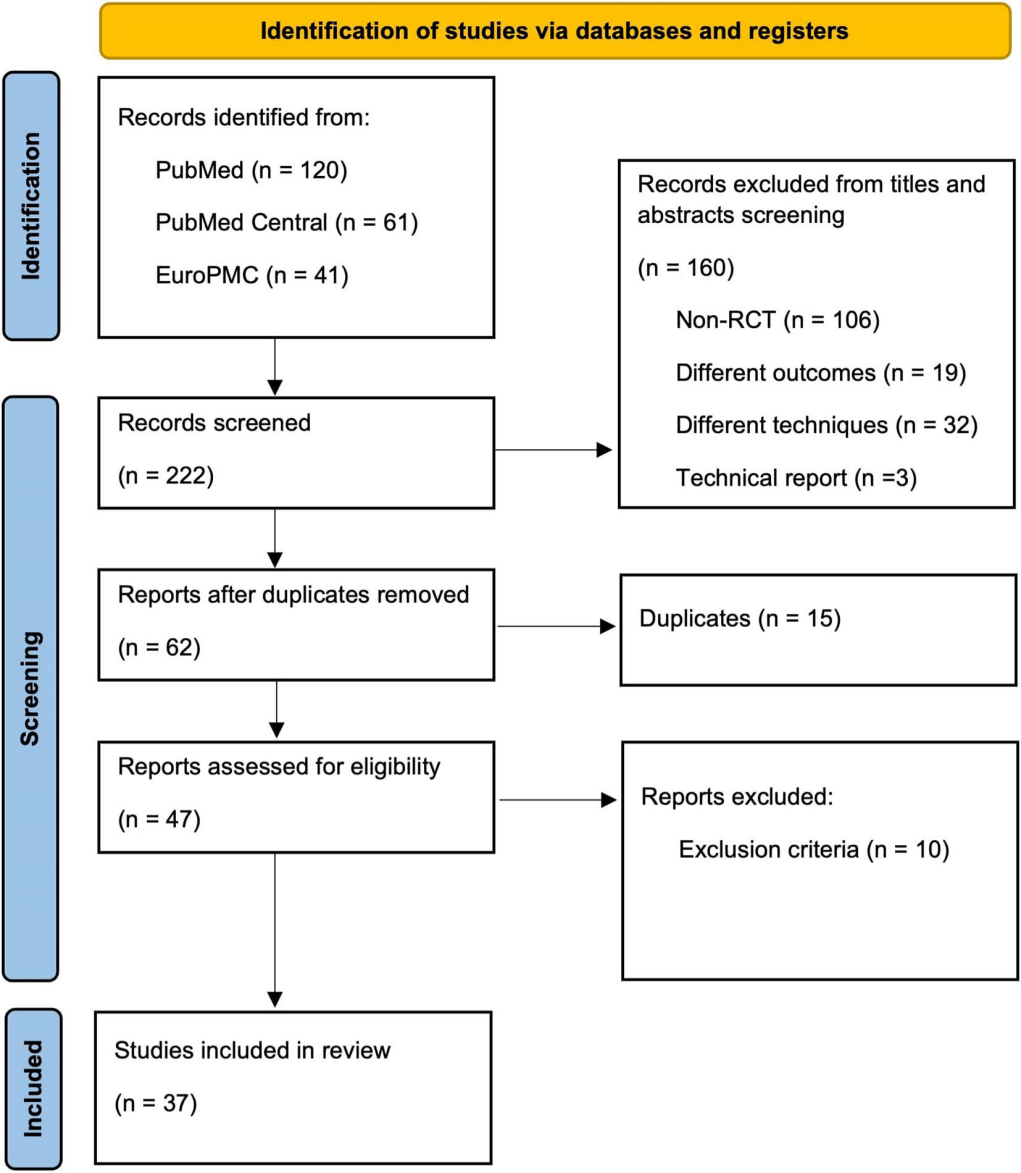
PRISMA flowchart. RISMA, Preferred Reporting Items for Systematic Reviews and Meta-Analyses; EuroPMC, Europe PubMed Central; RCT, randomised controlled trial.

We conducted systematic literature searches in three databases: PubMed (RRID:SCR_004846), PubMed Central (PMC) (RRID:SCR_004166), and Europe PubMed Central (EuroPMC) (RRID:SCR_005901) from December 2011 until December 2021. The latest search was conducted in January 2022. To filter the intended studies, combined keywords were used, but were not limited to, the following: “single-incision”, “single-port”, “single access”, “conventional”, “standard”, “multi-port”, “laparoscopic cholecystectomy”. Two researchers (RR and RVH) independently screened the titles and abstracts to find the eligible articles. Additional evaluation of references from eligible studies was also conducted to search for more potential articles. Full-text articles were then assessed independently according to the inclusion and exclusion criteria. This study is reported in line with the Preferred Reporting Items for Systematic Reviews and Meta-Analyses (PRISMA) guidelines ^63^ and the PRISMA diagram shows the strategy we employed during our study ( Figure 1).

### Data extraction and quality assessment

Two researchers (RR and RVH) independently conducted the data extraction process. An extraction form was developed to list information about the study, such as the study’s general information (title, authors, year of publication, study design), population characteristics, and outcomes measured. The data were then extracted through Covidence (RRID:SCR_016484). Conflicts in data abstraction were resolved by consensus and referring to the original article.

Two authors (RR and RVH) assessed the quality of each study involved in this review independently. The Cochrane Risk of Bias (RoB) 2.0 tool was used to evaluate the quality of clinical trial studies. This tool is comprised of five domains, including (1) bias arising from the randomization process; (2) bias due to deviations from intended interventions; (3) bias due to missing outcome data; (4) bias in measurement of the outcome; and (5) bias in the selection of the reported results. The final judgments of each domain were categorized as low risk, some concerns, or high risk. The summarized five domains of RoB were concluded by RR and RVH, whereas a discussion with AW resolved any discrepancies in the judgments to reach the final consensus.

### Statistical analysis

Meta-analysis was done using Review Manager 5.4 (Cochrane Collaboration) and Comprehensive Meta-Analysis version 3 software. The Mantel-Haenszel formula was used to obtain the risk ratio (RR) and 95% confidence interval (CI). At the same time, the Inverse Variance method was used to obtain the mean difference (MD), standardized mean difference (SMD), and standard deviation (SD). We used the random-effects model for all outcomes of interest in this study, regardless of heterogeneity. This meta-analysis assessed heterogeneity between studies by I-squared (I ^2^; inconsistency). The I ^2^ statistic with a value of <25% is considered a low degree of heterogeneity, 26-50% is a moderate degree of heterogeneity, and >50% is considered a high degree of heterogeneity. Funnel plot analysis was utilized to assess the qualitative risk of publication bias, while Egger’s regression method was used to evaluate the quantitative risk of publication bias.

## Results

### Study selection and study characteristics

The initial search generated 222 records, in which 37 RCTs were included for qualitative synthesis (systematic review) following the screening, removing duplication, and excluding of several studies ( Figure 1). ^14^^–^^50^ These final included RCTs yielded 2,129 and 2,392 patients who underwent SILC and CMLC. All patients had uncomplicated gall bladder diseases with ASA grade I-III and were eligible to undergo both techniques. The types of surgical ports ranged from conventional instruments to specific models. Three studies were double-blind RCTs, one was a single-blind RCT, and the remaining RCTs did not elaborate on the blinding method. A total of 10 studies were excluded due to including BMI >35 kg/m ^2^, age <18 years old, and retrospective study. ^51^^–^^60^ The full details of data characteristics are available in Table 1.

**Table 1.** Data characteristics.

Authors	Year	Type of port(s)		Sample size, n		Age, years, mean (SD)	Male sex, n (%)	BMI, kg/m ^2^, mean (SD)	ASA grade, mean (SD)
		SILC	CMLC	SILC	CMLC				
Arezzo *et al.* ^14^	2017	Singe-port device	10 mm port and two 2 mm port	263	278	48.3 (15.8)	NR	26.9 (4.1)	NR
Bingener *et al.* ^15^	2016	TriPort™ (Olympus)	Two 10 mm and two 5 mm port	55	55	48.4 (16.2)	21 (19.1%)	31.7 (6.1)	1.95 (0.5)
Borle *et al.* ^16^	2014	Conventional instruments	Two 11 mm and two 5 mm port	30	30	41.25 (13.09)	17 (28.3%)	23.2 (1.7)	NR
Brown *et al.* ^17^	2013	SILS ^®^ port (Covidien)	One 11 mm and three 5 mm port	40	39	45 (13.9)	18 (22.8%)	29.8 (6.0)	NR
Bucher *et al.* ^18^	2011	TriPort™ (Olympus)	Two 10 mm and two 5 mm port	75	75	46.12 (10.03)	NR	35.3 (2.5)	2 (0.3)
Cao *et al.* ^19^	2011	Conventional instruments	Two 10 mm and one 5 mm port	57	51	61.0 (4.9)	45 (41.7%)	28.3 (4.7)	1.85 (0.6)
Chang *et al.* ^20^	2015	SILS ^®^ port (Covidien)	10 mm port (Genicon)	50	50	50.3 (12.9)	39 (39%)	25.5 (5.5)	1.65 (0.5)
Deveci *et al.* ^21^	2013	SILS ^®^ port (Covidien)	Two 10 mm and one 5 mm port	50	50	41.4 (12.1)	12 (12%)	28 (5.02)	1.75 (0.5)
Ellatif *et al.* ^22^	2013	Conventional instruments	Two 10 mm and two 5 mm port	125	125	47.3 (10.9)	67 (26.8%)	28.2 (5.7)	1.55 (0.3)
Goel *et al.* ^23^	2016	Conventional instruments	NR	30	30	38.5 (7.8)	11 (18.3%)	NR	NR
Guo *et al.* ^24^	2015	Conventional instruments	Two 10 mm and one or two 5 mm port	138	414	43.9 (12.1)	137 (24.8%)	24.9 (2.7)	NR
Hajong *et al.* ^25^	2016	Conventional instruments	Two 10 mm and two 5 mm port	32	32	NR	12.50%	NR	NR
He *et al.* ^26^	2015	Conventional instruments	One 10 mm and two 5 mm port	100	100	40.6 (13.3)	99 (49.5%)	26.1 (7.1)	1.6 (0.5)
Ito *et al.* ^27^	2019	Single device (EZ ACCESS)	One 12 mm and three 5 mm port	58	53	57.4 (12.5)	47 (42.3%)	24.5 (3.7)	NR
Jorgensen *et al.* ^28^	2014	SILS ^®^ port (Covidien)	One 12 mm and two 5 mm port	60	60	45.1 (5.4)	NR	25.7 (1.6)	1.35 (0.5)
Justo-Janeiro *et al.* ^29^	2014	SILS ^®^ port (Covidien)	Two 10 mm and one 5 mm port	17	17	43.4 (16.4)	5 (14.7%)	27.8 (3.9)	1.5 (0.25)
Khorgami *et al.* ^30^	2014	Conventional instruments	One 10 mm and three 5 mm port	30	30	42.6 (11.8)	17 (28.3%)	27.3 (4.1)	NR
Klein *et al.* ^31^	2020	TriPort™ or TriPort+™ (Olympus)	Two 10 mm and one 5 mm port	98	95	47.2 (15.4)	64 (33.1%)	28.6 (6.1)	1.6 (0.3)
Koirala *et al.* ^32^	2019	Conventional instruments	Two 10 mm and two 5 mm port	100	100	41 (14.2)	41 (20.5%)	NR	NR
Lai *et al.* ^33^	2011	SILS ^®^ port (Covidien)	One 10 mm and three 5 mm port	24	27	53.1 (12.6)	19 (37.3%)	24.7 (2.9)	1.5 (0.5)
Leung *et al.* ^34^	2012	NR	NR	36	43	47.5 (19.2)	25.70%	28.5 (6.4)	NR
Lirici *et al.* ^35^	2011	TriPort™ (Olympus)	Two 12 mm and two 5 mm port	20	20	46.2 (13.2)	12 (30%)	24.8 (2.9)	1.9 (0.5)
Luna *et al.* ^36^	2013	SITRACC device	Two 10 mm and two 5 mm ports	20	20	NR	NR	NR	NR
Lurje *et al.* ^37^	2015	SILS ^®^ port (Covidien)	NR	48	48	46.0 (14.0)	62 (64.6%)	25.0 (4.0)	1.5 (0.3)
Noguera *et al.* ^38^	2013	SILS ^®^ port (Covidien)	One 1 mm and two 5 mm port	20	20	54.5 (7.1)	7 (17.5%)	29.0 (3.1)	1.6 (0.5)
Partelli *et al.* ^39^	2016	Single Site Laparoscopic Access System	Two 10 mm and two 5 mm port	30	29	44.8 (12.9)	22 (37.2%)	24.2 (3.4)	NR
Qu *et al.* ^40^	2019	Single port	Two 10 mm and two 5 mm port	49	42	46.4 (9.8)	41 (45.1%)	23.3 (2.7)	NR
Rizwi *et al.* ^41^	2014	NR	NR	100	100	41.7 (8.3)	84 (42%)	NR	NR
Saad *et al.* ^42^	2013	SILS ^®^ port (Covidien)	Two 10 mm and two 5 mm port	35	35	47.0 (15.6)	54 (77.1%)	25.4 (2.8)	1.6 (0.5)
Sasaki *et al.* ^43^	2012	SILS ^®^ port (Covidien)	One 12 mm and three 5 mm port	27	27	57.4 (13.2)	28 (51.8%)	24.7 (3.2)	NR
Solomon *et al.* ^44^	2012	SILS ^®^ port (Covidien)	One 11 mm and three 5 mm port	22	11	37.4 (3.8)	NR	31.7 (1.7)	NR
Subirana *et al.* ^45^	2021	TriPort+™ (Olympus)	Two 10 mm and two 5 mm port	37	36	38.1 (10.3)	9 (12.3%)	26.3 (3.8)	1.35 (0.3)
Sulu *et al.* ^46^	2015	SILS ^®^ port (Covidien)	Two 5 mm and two 2 mm port	30	30	46.3 (9.7)	21 (35%)	29.4 (5.0)	1.6 (0.5)
Ye *et al.* ^47^	2015	Single 5mm port	NR	100	100	NR	NR	NR	NR
Yilmaz *et al.* ^48^	2013	SILS ^®^ port (Covidien)	NR	43	40	49.7 (10.7)	61 (73.4%)	23.8 (3.6)	2.0 (0.5)
Zhao *et al.* ^49^	2016	Single Site Laparoscopic Access System	NR	50	50	48.1 (8.9)	32 (32%)	24.9 (3.8)	NR
Zheng *et al.* ^50^	2012	A single Tri-port (Advanced Surgical Concept)	Two 10 mm and one 5 mm port	30	30	45.2 (12.9)	29 (48.3%)	25.3 (3.8)	NR

SILC, single-incision laparoscopic cholecystectomy; CMLC, conventional multi-port laparoscopic cholecystectomy; ASA, American Society of Anesthesiologists; BMI, body mass index; SD, standard deviation; NR, not reported.

### Risk of bias in studies

**Figure 2.**
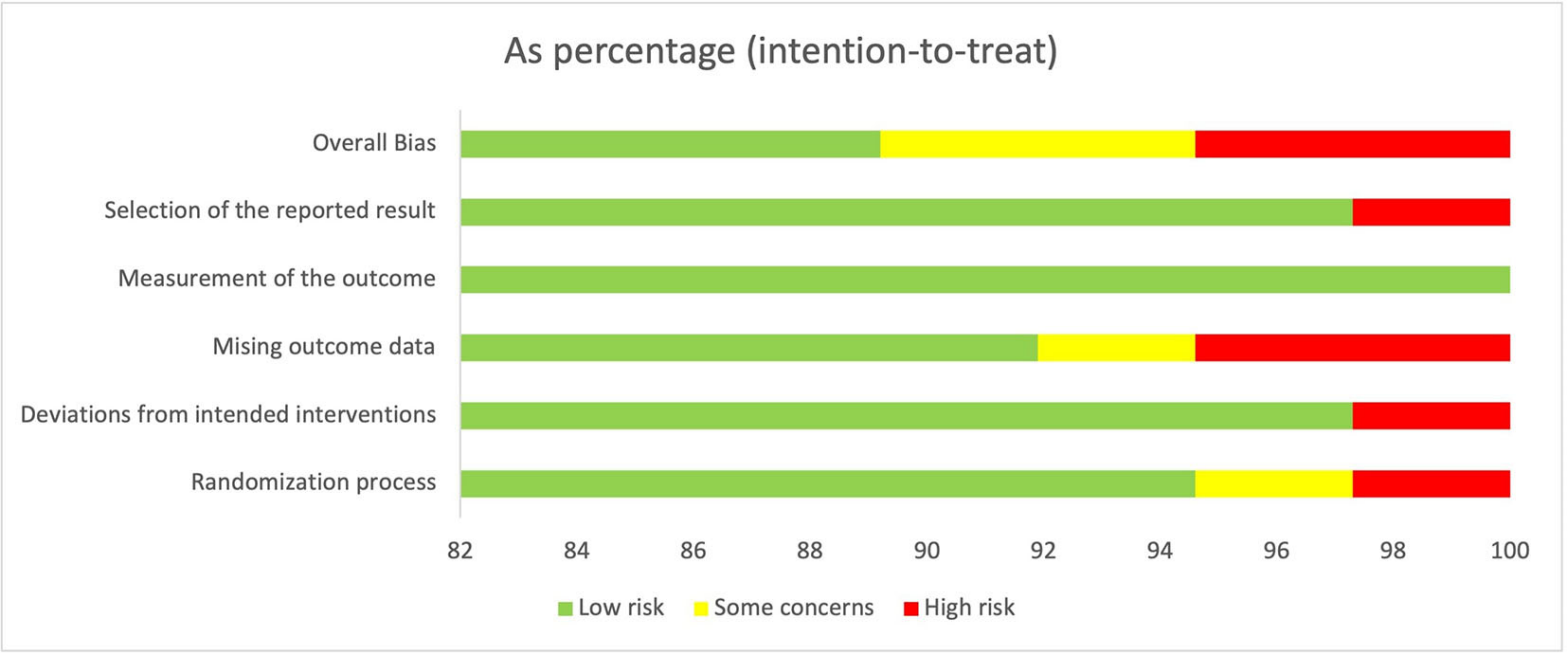
The overall judgment of included studies.

The Cochrane RoB 2.0 tool was applied to determine the quality of RCTs. A total of 33 studies were categorized as low risk of bias studies. Two studies were determined to have some concerns because the long-term follow-up results could not be fully concluded. The other two studies were evaluated for having a high risk of bias due to having a higher percentage of loss to follow-up and a high risk of false-positive results. The evaluation of RoB is summarized in Figure 2.

### Outcome effects

*Bile duct injury*

**Figure 3.**
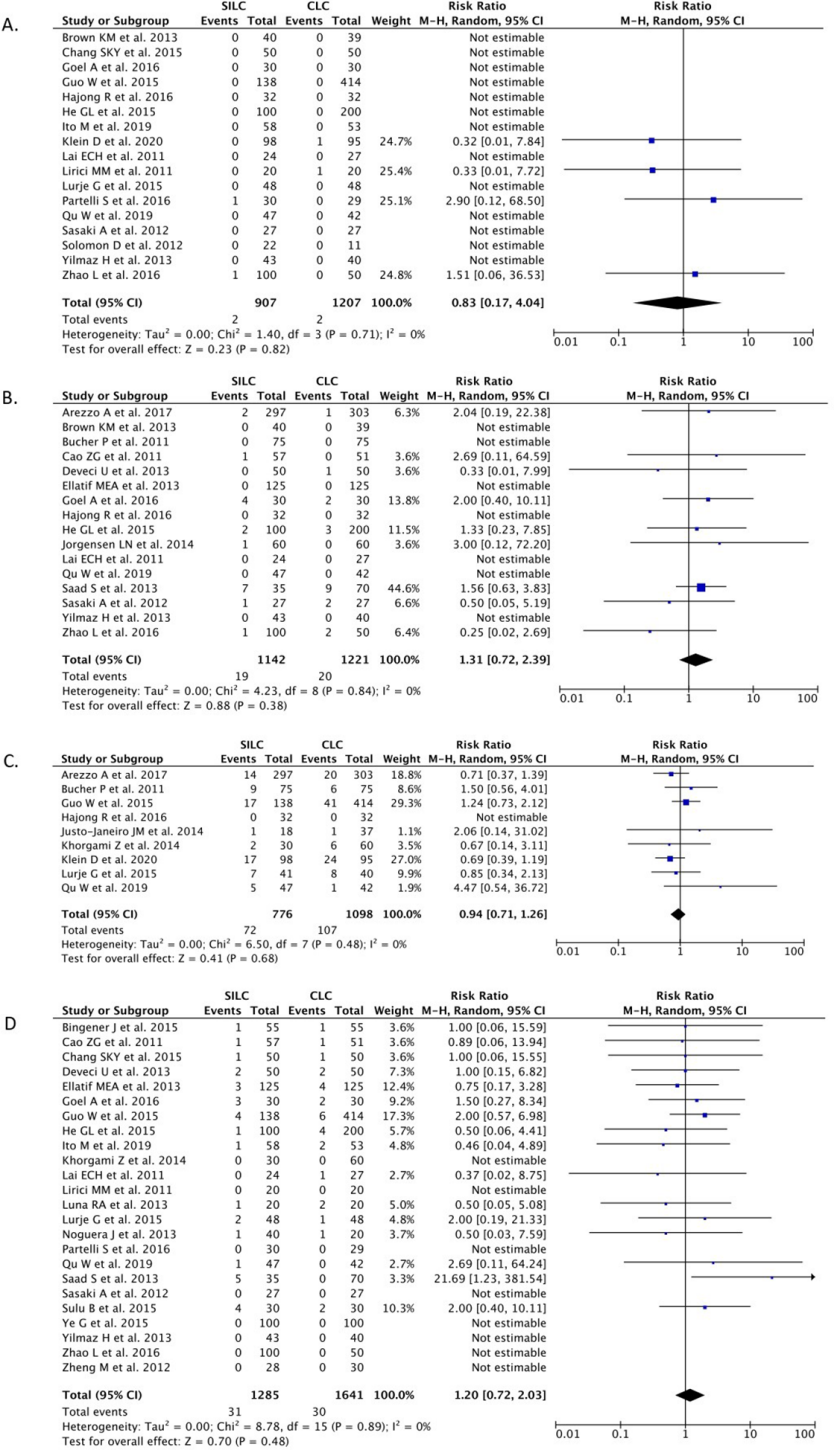
Forrest plots of SILC vs. CMLC. (A) Bile duct injury. (B) Bile leakage. (C) Gall bladder perforation. (D) Wound infection. SILC, single-incision laparoscopic cholecystectomy; CMLC, conventional multi-port laparoscopic cholecystectomy.

A total of 17 studies (n=2,114) reported the BDI outcome. Our pooled analysis showed that the SILC procedure did not differ from the CMLC procedure in terms of risk for BDI [RR 0.83 (95% CI 0.17–4.04), p=0.82, I ^2^=0%, random-effect modeling] ( Figure 3A).

*Bile leakage*

A total of 16 studies (n=2,363) reported on the bile leakage outcome. Our pooled analysis showed that the SILC procedure did not differ from the CMLC procedure in terms of risk for bile leakage incidence [RR 1.31 (95% CI 0.72–2.39), p=0.38, I ^2^=0%, random-effect modelling] ( Figure 3B).

*Gallbladder perforation*

Nine studies (n=1,874) reported on the gallbladder perforation outcome. Our pooled analysis showed that the SILC procedure did not differ from the CMLC procedure in terms of gallbladder perforation incidence [RR 0.94 (95% CI 0.71–1.26), p=0.68, I ^2^=0%, random-effect modelling] ( Figure 3C).

*Wound infection*

A total of 24 studies (n=2,926) reported on the wound infection outcome. Our pooled analysis showed that the SILC procedure did not change the risk of wound infection incidence when compared with the CMLC procedure [RR 1.20 (95% CI 0.72–2.03), p=0.48, I ^2^=0%, random-effect modelling] ( Figure 3D).

*Incisional hernia*

**Figure 4.**
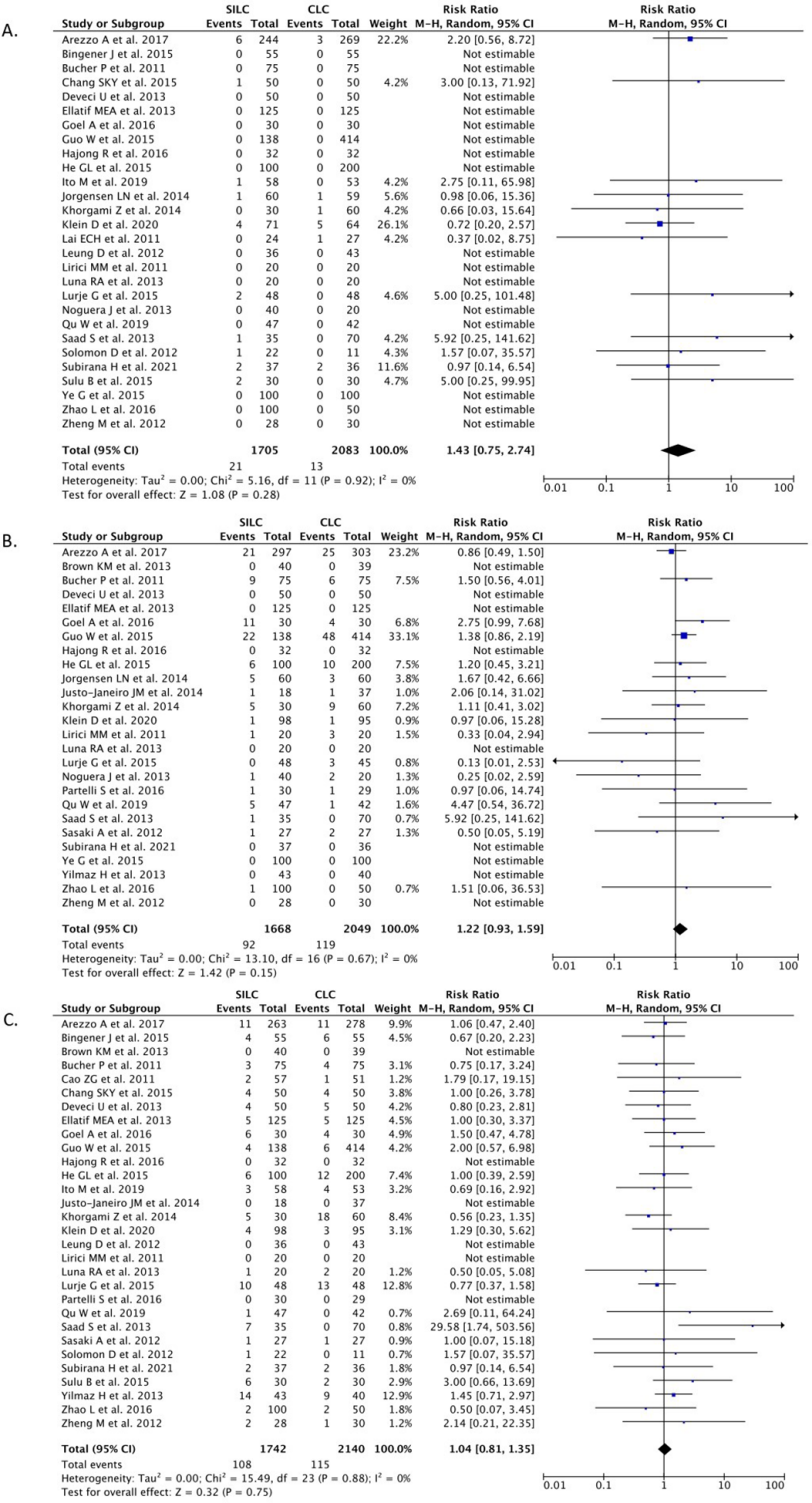
Forrest plots of SILC vs. CMLC. (A) Incisional hernia. (B) Total intra-operative complications. (C) Total post-operative complications. SILC, single-incision laparoscopic cholecystectomy; CMLC, conventional multi-port laparoscopic cholecystectomy.

A total of 28 studies (n=3,788) reported on the incisional hernia outcome. Our pooled analysis showed that the SILC procedure did not change the risk of incisional hernia incidence when compared with the CMLC procedure [RR 1.43 (95% CI 0.75–2.74), p=0.28, I ^2^=0%, random-effect modelling] ( Figure 4A).

*Total intra-operative complications*

A total of 26 studies (n=3,717) reported on the total intra-operative outcome. Our pooled analysis showed that the SILC procedure did not differ from the CMLC procedure in terms of the incidence of total intra-operative complications [RR 1.22 (95% CI 0.93–1.59), p=0.15, I ^2^=0%, random-effect modelling] ( Figure 4B).

*Total post-operative complications*

A total of 30 studies (n=3,882) reported on the total post-operative outcome. Our pooled analysis showed that the SILC procedure did not differ from the CMLC procedure in terms of the incidence of total post-operative complications [RR 1.04 (95% CI 0.93–1.59), p=0.15, I ^2^=0%, random-effect modelling] ( Figure 4C).

*Estimated blood loss*

**Figure 5.**
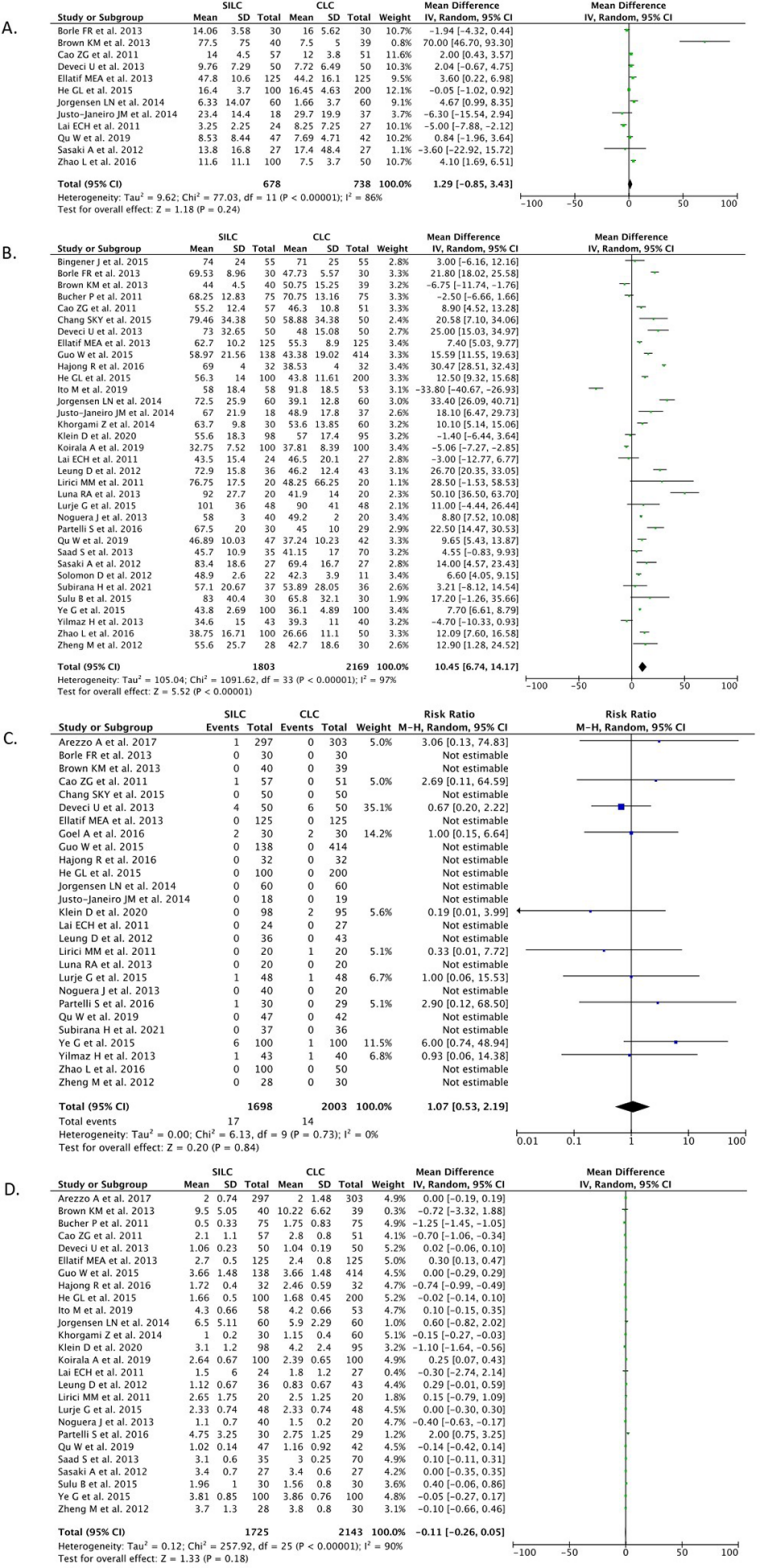
Forrest plots of SILC vs. CMLC. (A) Estimated blood loss. (B) Operation time. (C) Conversion to open surgery. (D) Hospital stay. SILC, single-incision laparoscopic cholecystectomy; CMLC, conventional multi-port laparoscopic cholecystectomy.

A total of 12 studies (n=1,416) reported on the estimated blood loss outcome. Our pooled analysis showed that the SILC procedure did not differ from the CMLC procedure in terms of estimated blood loss volume [MD 1.29 (95% CI -0.85, 3.43), p=0.24, I ^2^=86%, random-effect modelling] ( Figure 5A).

*Operation time*

A total of 34 studies (n=3,972) reported on the operation time outcome. Our pooled analysis showed that the SILC procedure significantly increased the length of operation time when compared with the CMLC procedure [MD 10.45 (95% CI 6.74, 14.17), p<0.00001, I ^2^=97%, random-effect modelling] ( Figure 5B).

*Conversion to open cholecystectomy*

A total of 27 studies (n=3,701) reported on the conversion to open cholecystectomy outcome. Our pooled analysis showed that the SILC procedure did not differ from the CMLC procedure in terms of conversion to open cholecystectomy rate [RR 1.07 (95% CI 0.53–2.19), p=0.84, I ^2^=0%, random-effect modelling] ( Figure 5C).

*Length of hospital stay*

A total of 26 studies (n=3,868) reported on the length of hospital stay outcome. Our pooled analysis showed that the SILC procedure did not differ from the CMLC procedure in terms of length of hospital stay [MD -0.11 (95% CI -0.26, 0.05), p=0.18, I ^2^=90%, random-effect modelling] ( Figure 5D).

*VAS at four hours post-operation*

**Figure 6.**
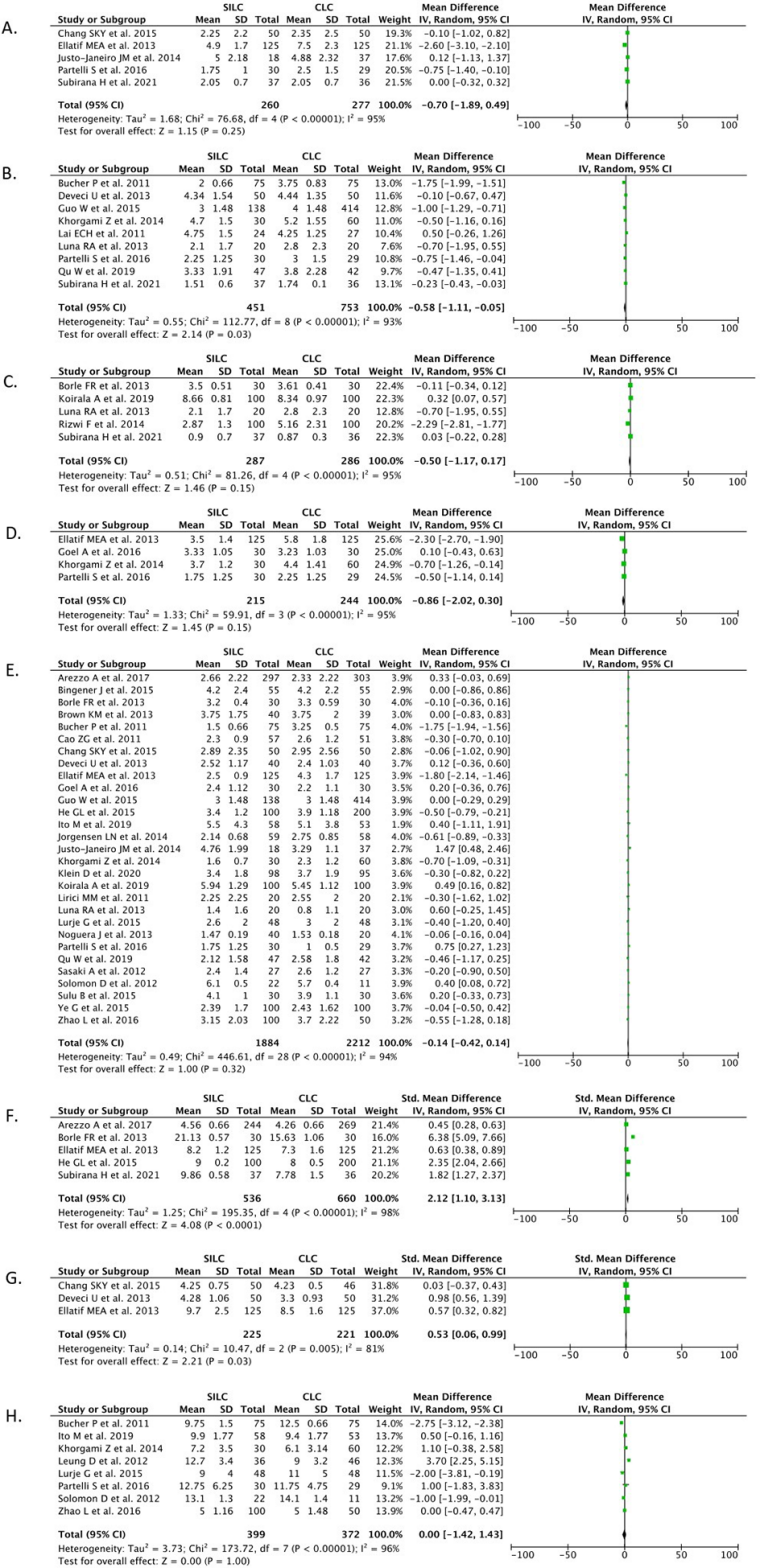
Forrest plots of SILC vs. CMLC. VAS at (A) 4, (B) 6, (C) 8, (D) 12 and 24 hours post-operation. Cosmesis at (F) one month and (G) six months post-operation. (H) Days to return to work. SILC, single-incision laparoscopic cholecystectomy; CMLC, conventional multi-port laparoscopic cholecystectomy; VAS, visual analog score.

Five studies (n=537) reported on VAS at the four-hour post-operative observation. Our pooled analysis showed that the SILC procedure did not offer benefits in VAS at four hours post-operation when compared with the CMLC procedure [MD -0.70 (95% CI -1.89, 0.49), p=0.25, I ^2^=95%, random-effect modelling] ( Figure 6A).

*VAS at six hours post-operation*

Nine studies (n=1,204) reported on VAS at the six-hour post-operative observation. Our pooled analysis showed that the SILC procedure offered benefits in reducing the VAS at six hours post-operation when compared with the CMLC procedure [MD -0.58 (95% CI -1.11, -0.05), p=0.03, I ^2^=93%, random-effect modelling] ( Figure 6B).

*VAS at eight hours post-operation*

Five studies (n=573) reported on VAS at the eight-hour post-operative observation. Our pooled analysis showed that the SILC procedure did not offer benefits in VAS at eight hours post-operation when compared with the CMLC procedure [MD -0.50 (95% CI -1.17, 0.17), p=0.15, I ^2^=95%, random-effect modelling] ( Figure 6C).

*VAS at 12 hours post-operation*

Four studies (n=459) reported on VAS at the 12-hour post-operative observation. Our pooled analysis showed that the SILC procedure did not offer benefits in VAS at 12 hours post-operation when compared with the CMLC procedure [MD -0.86 (95% CI -2.02, 0.30), p=0.15, I ^2^=95%, random-effect modelling] ( Figure 6D).

*VAS at 24 hours post-operation*

A total of 29 studies (n=4,096) reported on VAS at the 24-hour post-operative observation. Our pooled analysis showed that the SILC procedure did not offer benefits in VAS at 24 hours post-operation when compared with the CMLC procedure [MD -0.14 (95% CI -0.42, 0.14), p=0.32, I ^2^=94%, random-effect modelling] ( Figure 6E).

*Cosmesis at one month*

Five studies (n=1,196) reported on the outcome of cosmesis one-month post-operation. Our pooled analysis showed that the SILC procedure offered benefits in increasing the cosmesis score one-month post-operation when compared with the CMLC procedure [SMD 2.12 (95% CI 1.10, 3.13), p<0.0001, I ^2^=98%, random-effect modelling] ( Figure 6F).

*Cosmesis at six months*

Three studies (n=446) reported on the outcome of cosmesis six months post-operation. Our pooled analysis showed that the SILC procedure offered benefits in increasing the cosmesis score at six months post-operation compared with the CMLC procedure [SMD 0.53 (95% CI 0.06, 0.99), p=0.03, I ^2^=81%, random-effect modelling] ( Figure 6G).

*Days to return to work*

Eight studies (n=771) reported on the days to return to work outcomes. Our pooled analysis showed that the SILC procedure did not differ from the CMLC procedure in terms of the number of days it took to return to work [MD 0.00 (95% CI -1.42, 1.43), p=1.00, I ^2^=96%, random-effect modelling] ( Figure 6H).

*Meta-regression*

Meta-regression was performed to identify risk factors that influence the relationship between SILC procedure and statistically significant outcomes, consisting of the operation time, VAS at six hours post-operation, cosmesis score at one-month post-operation, and cosmesis score at six months post-operation. The results of the meta-regression analyses can be found as *Extended data.* ^63^ Our meta-regression revealed that variability in those outcomes in patients who underwent the SILC procedure compared with the CMLC procedure was explained by known patient factors associated with predictors of abdominal surgery outcomes. From our meta-regression analysis, it was revealed that the length of operation time in patients who underwent the SILC procedure compared with the CMLC procedure was not significantly influenced by age (p=0.1133), sex (p=0.1936), BMI (p=0.4407), and ASA score (p=0.0557). In terms of VAS at six hours post-operation, a statistically significant association was present for BMI (beta coefficient: -0.1120; 95% CI: -0.2161, -0.0079; p=0.0350). However, other factors such as age (p=0.7800), sex (p=0.8660), and ASA score (p=0.0976) did not significantly affect the relationship between the SILC procedure compared with the CMLC procedure on the VAS at six hours post-operation. Meanwhile, in terms of cosmesis at one-month post-operation, a statistically significant association was also present for BMI (beta coefficient: -1.2241; 95% CI: -1.7181, -0.7302; p *<*0.0001). Our meta-regression also revealed that cosmesis at one-month post-operation was not significantly influenced by age (p=0.2866), and sex (p=0.9090). Lastly, for cosmesis at six months post-operation, the meta-regression analysis could not be performed as there were too many included studies in the analysis.

*Publication bias*

We used Funnel plot analysis for the assessment of publication bias in each outcome of interest. This analysis showed a relatively symmetrical inverted plot for all outcomes of interest in this study, indicating no publication bias. ^63^ Furthermore, the Egger regression test results were also not statistically significant for all outcomes of interest, confirming the results from funnel plot analysis in which no sign of publication bias was found ( Table 2).

**Table 2.** Summary of publication bias analysis.

Outcomes	Funnel-plot	Egger’s test
Bile duct injury	Symmetrical	p=0.93632
Bile leakage	Symmetrical	p=0.42522
Gallbladder perforation	Symmetrical	p=0.28977
Wound infection	Symmetrical	p=0.62726
Incisional hernia	Symmetrical	p=0.27176
Total intra-operative complications	Symmetrical	p=0.71848
Total post-operative complications	Symmetrical	p=0.14619
Estimated blood loss	Symmetrical	p=0.57188
Operation time	Symmetrical	p=0.82622
Conversion to open cholecystectomy	Symmetrical	p=0.54945
Length of hospital stay	Symmetrical	p=0.38360
VAS at four hours post-operation	Symmetrical	p=0.83937
VAS at six hours post-operation	Symmetrical	p=0.59750
VAS at six hours post-operation	Symmetrical	p=0.28533
VAS at 12 hours post-operation	Symmetrical	p=0.18789
VAS at 24 hours post-operation	Symmetrical	p=0.47530
Cosmesis at one-month post-operation	Symmetrical	p=0.08651
Cosmesis at six months post-operation	Symmetrical	p=0.95798
Days to return to work	Symmetrical	p=0.95410

VAS, visual analog score.

## Discussion

The current ‘gold standard’ for cholecystectomy is laparoscopic cholecystectomy. This well-known technique has superseded the open approach for routine cholecystectomy since the 1990s. Multiple studies have demonstrated that conventional laparoscopic (three or four ports) is feasible, safe, and favourable for cholecystectomy. ^61^ Advancements in surgical technology are accelerating; thus, the single incision approach was introduced in 1997 to improve post-operative pain and cosmetic results. At present, SILC and CMLC are the current options for cholecystectomy with various considerations, and many RCTs showed the strengths and limitations of each technique, although the conclusion remains contentious. ^1^

A recent systematic review by Lyu *et al*., ^12^ stated that SILC did not offer advantages over CMLC. The overall results of our study showed that SILC improved post-operative pain and cosmetic results with no higher incidence of perioperative complications, blood loss, conversion to open cholecystectomy, extended hospital stay, and days return to work compared to CMLC. On the other hand, longer operation time is still a major issue of SILC.

Perioperative complications are the current issue and remain disputable in every study. Evers *et al*., ^7^ in 2017 demonstrated that SILC was inferior to CMLC [RR 3.00 (95% CI 1.05–8.58)]. Another systematic review conducted by Hall *et al*., ^11^ further supported the inferiority of SILC regarding overall complications. Meanwhile, our study showed that biliary duct injuries, bile leakage, gall bladder perforation, wound infection, and incisional hernia did not differ between SILC and CMLC. These statements are parallel with other systematic reviews. ^9^^,^^13^^,^^62^ Our suppositions are that the enhanced knowledge and advancement in single incision port have minimized the potential complications. Hence, SILC is considered as feasible as CMLC.

The learning curve that remains to be overcome for SILC is operation time. Our study demonstrated a longer time in SILC with a significant p-value. None of the other systematic reviews showed a quicker time in SILC than CMLC. ^7^^,^^9^^,^^11^^,^^13^ Many aspects can hinder the operation time of SILC, including the peculiar technique, type of instruments, camera angle perspective, and instruments crossing. These issues can be overcome by surgical experience and the development of advanced instruments. Thus, the operation time may be equal in both approaches.

Post-operative pain was calculated using the VAS at four time points (4, 6, 8, 12 and 24 hours). Our study showed a significant pain reduction at pain score six hours post-operation; however, VAS at 4, 8, 12 and 24 hours did not differ in both groups. The studies were done by Hall *et al*., ^11^ and Arezzo *et al*., ^9^ stated the VAS did not differ significantly. Meanwhile, Lirici *et al*., ^62^ demonstrated an improvement in VAS after day one, although the pain score on day one did not show any differences between SILC and CMLC. On the other hand, Evers *et al*., ^7^ revealed a superior VAS outcome in the SILC group at 24 hours. The opposite outcomes were reported by Lyu *et al*.,, ^12^ in which SILC remained inferior for post-operative pain reduction at 6, 8, 12 and 24 hours. The heterogeneity of VAS was influenced by many elements, including the type of anaesthetic drugs, length of incision, and psychological factors. Thus, our study reported a high degree of heterogeneity.

In terms of cosmesis, the superiority of SILC was undoubtedly observed. Our study reported better cosmetic results at both the one-month and six-month post-operative outcomes. Multiple studies have also supported our results. ^7^^,^^9^^,^^11^^,^^62^ The advantage of having a single incision at the umbilical creates a seamless scar, increasing the patient’s satisfaction. The definition of cosmesis was varied in every study; consequently, heterogeneity was high.

Our study did not find any differences between SILC and CMLC concerning blood loss, conversion to open surgery, hospital stay, and days to return to work. Supporting studies also stated equivalent results, which SILC did not enhance the outcome of those categories. ^7^^,^^9^^,^^11^^,^^62^

The limitations regarding this study are that we only included studies in English, a high degree of heterogeneity of several aspects, numerous RCTs with a high risk of bias, and inconsistent definitions of every outcome measured. We suggest an equal scoring for pain and cosmesis score to minimize the heterogeneity, as well as a more precise definition of perioperative complications and operation time to unify the outcome measurement. Hence, large-scale, double-blinded, well-designed RCTs are recommended.

## Conclusions

SILC is a safe, feasible and favourable approach. In terms of pain reduction and cosmetic results, SILC offers promising results. Thus, SILC may be an option for cholecystectomy depending on the surgeon’s experience.

## Data availability

### Underlying data

All data underlying the results are available as part of the article and no additional source data are required.

### Extended data

Zenodo: Single-incision laparoscopic cholecystectomy versus conventional multi-port laparoscopic cholecystectomy: A systematic review, meta-analysis, and meta-regression of randomized controlled trials. https://doi.org/10.5281/zenodo.6416832. ^63^

This project contains the following extended data:

- ROB2_IRPG_beta_v9_Sysrev.xlsm (dataset)
- Supplementary Figure 1.pdf
- Supplementary Figure 2.pdf
- Supplementary Figure 3.pdf
- Supplementary Figure 4.pdf
- Supplementary Table 1.docx
- Search Strategy.docx

## Reporting guidelines

Zenodo: PRISMA checklist for ‘Single-incision laparoscopic cholecystectomy versus conventional multi-port laparoscopic cholecystectomy: A systematic review, meta-analysis, and meta-regression of randomized controlled trials.’ https://doi.org/10.5281/zenodo.6416832. ^63^

Data are available under the terms of the Creative Commons Attribution 4.0 International license (CC-BY 4.0).
