# Supplementary material for: Single-incision laparoscopic cholecystectomy versus conventional multi-port laparoscopic cholecystectomy: A systematic review, meta-analysis, and meta-regression of randomized controlled trials
Source: F1000Res. 2024 Nov 18;11:754. Originally published 2022 Jul 6. [Version 2] doi: 10.12688/f1000research.122102.2 (PMC11809676; doi:10.12688/f1000research.122102.2)
Supplement: Peer review history

# Reviewer report: Reviewer response for version 2

**DOI:** 10.5256/f1000research.174548.r345779

**Published:** 2024-12-18

**Authors:** Islam Korayem

**Affiliations:** 1 Alexandria University, Alexandria, Alexandria Governorate, Egypt

Thank you for giving me the chance to review this manuscript. Comparing different approached of laparoscopic cholecystectomy remains to be a hot topic with abundant contributions and debates.

 Overall, the manuscript is well written in a proper manner both scientifically and linguistically.

 There are, however, some points that require further attention and revision by the authors to improve the current version of the manuscript.

 - Conventional LC always refers to the standard 4-port LC performed with two 10/12 mm ports and two 5mm ports. Any modification of this pattern as reduced port size or number should not be considered conventional.

 Since the manuscript included studies comparing SILC to 4-port, 3-port, 2-port, or reduced-port size (3mm and 1mm), thus placing those as conventional is not accurate.

 Accordingly, I recommend the authors to omit the word "conventional" from the manuscript title as well as the group designation that is compared to SILC group.

 Using the term "Multiport LC (MPLC)" will be more descriptive as it will encompass all forms of multiport LC using any numbers and sizes of ports (including the conventional/standard LC).

 - Always add "respectively" to the end of the statement whenever you mention the 2 groups and their numbers following them to ensure the orderly fashion of the displayed numbers related to each the groups. This should be followed in the Abstract and the Results sections to look as such: *(2129 and 2392 patients were included in the SILC and MPLC groups, **respectively**).*

 - The heterogeneity of the MPLC group as illustrated in Table 1 should be addressed as a potential form of bias since such standard group is not the same across the included studies.

 - The PRISMA flowchart needs to be modified as follows:

 1- The first 2 boxes need to be merged into 1 box, where the total number of studies identified upon online search is 222 which is broken down to 120 from PubMed, 61 from PMC, and 41 from EuroPMC.

 The horizontal arrow going toward the excluded box should come out from the vertical downward arrow and Not from the box.

 2- The next box should be the remaining studies to be screened after exclusion which will be 62 studies.

 The arrow going downward from it should divide to a horizontal arrow which illustrates the exclusion of 15 duplicate studies.

 3- The next box should be the remainder after exclusion of duplicates in the above step, which will be 47 studies.

 The arrow going vertically downwards from this third box will have another horizontal arrow going to the excluded 10 studies based on exclusion criteria defined.

 4- A final box of 37 studies included in the analysis should be the end of this chart

 - In the Methodology, defining pain assessment using VAS should also include the type of the analgesic used, the dosing, and the frequency of administration across all the studies to ensure a transparent comparison of both groups.

  - In the discussion section, the first paragraph is repeated twice (lines 1-7, and lines 7-14). Delete the any group of the repeated lines.

 - The beginning of the paragraph should be modified as follows: ( *The gold standard treatment for **gallstone disease** is laparoscopic cholecystectomy).*

 - The conclusion needs to better highlight the results arising from this study.

Are the rationale for, and objectives of, the Systematic Review clearly stated?

Yes

Is the statistical analysis and its interpretation appropriate?

Yes

If this is a Living Systematic Review, is the ‘living’ method appropriate and is the search schedule clearly defined and justified? (‘Living Systematic Review’ or a variation of this term should be included in the title.)

Not applicable

Are sufficient details of the methods and analysis provided to allow replication by others?

Yes

Are the conclusions drawn adequately supported by the results presented in the review?

Partly

Reviewer Expertise:

Hepatobiliary and Pancreatic Surgery, Laparoscopic Abdominal Surgery, Liver resection, Liver Transplantation, Hernia Surgery, Colorectal Surgery, Gastrointestinal Surgery, Abdominoplasty Surgery

I confirm that I have read this submission and believe that I have an appropriate level of expertise to confirm that it is of an acceptable scientific standard, however I have significant reservations, as outlined above.

# Reviewer report: Reviewer response for version 2

**DOI:** 10.5256/f1000research.174548.r341301

**Published:** 2024-11-21

**Authors:** Yunushan Furkan Aydoğdu

**Affiliations:** 1 Department of General Surgery, Bandırma Training and Research Hospital, Balıkesir, Turkey

Dear Author

 In general, the requested revisions were made, but the article suggestions in the first revision was not added. Adding it will increase the development and acceptability of the article. It is also thought to increase its readability.

Are the rationale for, and objectives of, the Systematic Review clearly stated?

Yes

Is the statistical analysis and its interpretation appropriate?

Yes

If this is a Living Systematic Review, is the ‘living’ method appropriate and is the search schedule clearly defined and justified? (‘Living Systematic Review’ or a variation of this term should be included in the title.)

Not applicable

Are sufficient details of the methods and analysis provided to allow replication by others?

Yes

Are the conclusions drawn adequately supported by the results presented in the review?

Partly

Reviewer Expertise:

General Surgery

I confirm that I have read this submission and believe that I have an appropriate level of expertise to confirm that it is of an acceptable scientific standard, however I have significant reservations, as outlined above.

# Reviewer report: Reviewer response for version 1

**DOI:** 10.5256/f1000research.134047.r335341

**Published:** 2024-11-06

**Authors:** Yunushan Furkan Aydoğdu

**Affiliations:** 1 Department of General Surgery, Bandırma Training and Research Hospital, Balıkesir, Turkey

General Evaluation

 Subject and Objective: The study focused on the comparison of SILC and CMLC techniques. The purpose of the research is clearly defined and targets existing gaps in the literature. This draws the reader's attention.

 Methods: The methods section clearly describes the systematic review and meta-analysis processes. A specific framework is established with PICOS criteria. The search strategy and study selection process are described in detail.

 Results: The results section presented the analyzed data in a systematic way. Statistical data are well summarized, but could be strengthened with more visual support (graphs or tables).

 Discussion: The discussion section provides a good overview of the relevance of the findings to the existing literature. The advantages and disadvantages of SILC are clearly discussed. However, some sentences may need to be simplified to improve fluency.

 Recommendations

 Expansion of Limitations: The limitations section could be enriched by adding more detail. For example, more emphasis could be placed on the potential impacts of the language limitation in the study.

 Summarizing the Results: At the end of the study, it may be useful to provide a brief summary of the main findings and recommendations for future research. This makes it easier for readers to remember the main points.

 References and Citations: The use of references in more contexts throughout the study may increase the credibility of the text. (As a suggestion regarding the study I am reviewing, I would like to state that the article by Aydoğdu et al. (2024) titled “Minimally invasive approach in a rare emergency surgery, gallbladder perforation” should also be included in the evaluation. This study highlights the efficacy of minimally invasive approaches for a rare surgical emergency such as gallbladder perforation. )

 Conclusion

 Overall, the paper is well structured and provides important insights on the topic.

Are the rationale for, and objectives of, the Systematic Review clearly stated?

Yes

Is the statistical analysis and its interpretation appropriate?

Yes

If this is a Living Systematic Review, is the ‘living’ method appropriate and is the search schedule clearly defined and justified? (‘Living Systematic Review’ or a variation of this term should be included in the title.)

Not applicable

Are sufficient details of the methods and analysis provided to allow replication by others?

Yes

Are the conclusions drawn adequately supported by the results presented in the review?

Partly

Reviewer Expertise:

General Surgery

I confirm that I have read this submission and believe that I have an appropriate level of expertise to confirm that it is of an acceptable scientific standard, however I have significant reservations, as outlined above.

# Response

**Published:** 2024-11-07

**Authors:** Reno Rudiman

**Affiliations:** Padjadjaran University, Indonesia, Indonesia

Dear Dr. Yunushan Furkan Aydoğdu,

 Thank you for your insightful recommendations on our manuscript. Therefore, we will proceed to revise based on the details you have provided.

 Best regards

 Rudiman

# Reviewer report: Reviewer response for version 1

**DOI:** 10.5256/f1000research.134047.r155047

**Published:** 2024-02-07

**Authors:** Vicky Sumarki Budipramana, Andro Pramana Witarto

**Affiliations:** 1 Department of Surgery, Premier Surabaya Hospital, Surabaya, Indonesia; 2 Airlangga University, Surabaya, East Java, Indonesia

- In Fig. 1 regarding PRISMA flowchart, section “Reports excluded: Exclusion criteria (n = 10)” were not clearly detailed into some points and the number of the excluded studies in each of the exclusion criteria. It will be better to input the detailed information to avoid misunderstandings among readers regarding the exclusion criteria.
- In I ^2^ statistic’s cut-off values, the authors stated three categories: <25%, 26-50%, and >50%. “26-50%” should have been “25-50%”, shouldn't it? Please correct the written cut-off to prevent misinterpretation if the I ^2^ = 25%.
- The figure legends of Fig. 3 to Fig. 6 should have been clearly stated regarding the classification of the outcomes. Why were the figures separated into Fig. 3 to Fig. 6? Since the outcomes should have been only classified into three (feasibility, safety, and surgical outcome). This should also have gone to the “Outcome effect” section in the results. Please clasify the outcomes in the results section based on the primary goal of this study in order for the readers to understand the outcomes easily.
- Please explain the novelty of this study as compared with reference No. 12 since it also carries the similar title to the current study.
- In the discussion section, in paragraph 6, the authors are encouraged to add an explanation regarding the fact that cosmesis is influenced by BMI based on the meta-regression analysis. This is an interesting finding that should have been revealed in the discussion section.

Are the rationale for, and objectives of, the Systematic Review clearly stated?

Yes

Is the statistical analysis and its interpretation appropriate?

Yes

Are sufficient details of the methods and analysis provided to allow replication by others?

Yes

Are the conclusions drawn adequately supported by the results presented in the review?

Yes

Reviewer Expertise:

Gastroentestinal surgery

We confirm that we have read this submission and believe that we have an appropriate level of expertise to confirm that it is of an acceptable scientific standard.
